# Methyltransferase gene-mediated antiproliferative effect of 1,25(OH)_2_D_3_ on neuroblastoma cells

**DOI:** 10.1590/1806-9282.20241269

**Published:** 2025-06-16

**Authors:** Okan Sancer

**Affiliations:** 1 Süleyman Demirel University, Innovative Technologies Application and Research Center, Genetic Research Unit – Isparta, Turkey.; 2 Süleyman Demirel University, Faculty of Medicine, Department of Medical Biology – Isparta, Turkey.

**Keywords:** Calcitriol, DNMT3A, DNMT3B, PTEN, SH-SY5Y

## Abstract

**OBJECTIVE:**

1,25-Dihydroxyvitamin D_3_ is the body's active form of vitamin D and is also known as calcitriol. It has an effect on the body's immune system and is involved in the regulation of cell growth, differentiation, and apoptosis.

**METHODS:**

The effect of 1,25-Dihydroxyvitamin D_3_ on the viability of human neuroblastoma human neuroblastoma cell line cells was investigated by 3-(4,5-Dimethylthiazol-2-yl)-2,5-Diphenyltetrazolium Bromide assay, the effect on migration was investigated by wound healing assay, and methyltransferase genes (DNA methyltransferase 3 alpha and DNA methyltransferase 3 beta) and phosphatase and tensin homolog gene expression levels were investigated by real time polymerase chain reaction method.

**RESULTS:**

As a result of the 3-(4,5-Dimethylthiazol-2-yl)-2,5-Diphenyltetrazolium Bromide assay, 1,25-Dihydroxyvitamin D_3_ IC_50_:265.6 nM was found. It was observed that 100 and 265.6 nM applied to human neuroblastoma cell line cells had an antiproliferative effect. In addition, DNA methyltransferase 3 alpha and DNA methyltransferase 3 beta genes, which are effective as methyltransferases, caused a change in expression levels (p<0.05), while phosphatase and tensin homolog expression levels did not change (p>0.05).

**CONCLUSION:**

1,25-Dihydroxyvitamin D_3_ showed antiproliferative, antimigratory activity on neuroblastoma human neuroblastoma cell line cells and decreased the expression levels of genes involved in methylation, which may be evidence of its anticancer effect.

## INTRODUCTION

Vitamins are essential for a wide range of biochemical functions and are either insufficiently synthesised or not synthesised at all by the body, so they are obtained from the diet. Vitamin D is a group of sterols with hormone-like functions. It is classified as a fat-soluble vitamin. The active metabolite of vitamin D is 1,25-dihydroxy vitamin D_3_ [1,25(OH)_2_D_3_] (Calcitriol)^1^. It binds to cytosolic receptors found in the intestinal, muscle, and haematopoietic cells, as well as in some other tissues such as osteocytes and the brain. It is then transported to the cell nucleus, where it interacts with DNA and is involved in the control of more than 900 gene expressions. Furthermore, vitamin D plays a role in numerous pathological and physiological processes, including calcium metabolism, bone mineralisation, cancer, immune modulation, cardiovascular disease, and metabolic syndrome^2^. Furthermore, the biological properties of vitamin D include regulation of cell proliferation and increased cell differentiation, effects on apoptosis, and interactions with the epigenome on multiple levels^3^. Vitamin D is known to alter DNA methylation at the promoter of certain genes^3^.

In mammals, the process of DNA methylation involves the covalent transfer of a methyl group to the C-5 position of the cytosine ring in the CpG dinucleotide region of DNA by DNA methyltransferase. The genome region with the highest density of CpG dinucleotide repeats is known as a CpG island, which spans 1,000–1,500 base pairs in length^4^. There is evidence that 1,25-D_3_ is capable of inducing DNA demethylation; however, the mechanisms underlying the effect of 1,25-D_3_ on DNA methylation remain unclear. Nevertheless, in certain instances, demethylation has been observed to occur within a timeframe of 1–4 h, indicating the potential involvement of an active process. Notably, only early-stage tumours, but not late-stage tumours, demonstrated a correlation between vitamin D intake and reduced methylation^3^.

In human cancer cells, DNA hypermethylation mainly includes promoter hypermethylation of the tumour suppressor gene (TSG) and promoter hypermethylation of the human DNA mismatch repair system gene^5^.

In conclusion, alterations in DNA methylation may play a significant role in the expression of genes and the disruption of genomic integrity and may be involved in the development and progression of diseases. The aim of the present study was to investigate the impact of 1,25-dihydroxyvitamin D_3_ [1,25(OH)_2_D_3_] on methylation mechanisms in the Human neuroblastoma cell line (SH-SY5Y) cell line.

## METHODS

### Cell line

The human neuroblastoma cell line, SH-SY5Y (ATCC, VA, USA), was incubated with Dulbecco's Modified Eagle Medium (Capricorn, Germany) supplemented with 10% foetal bovine serum (Sigma-Aldrich, USA) and 100 IU/mL penicillin, 10 μg/mL streptomycin (Sigma-Aldrich, USA).

### 3-(4,5-Dimethylthiazol-2-yl)-2,5-Diphenyltetrazolium Bromide assay

This assay was performed according to Pedron et al^6^. Briefly, a 96-well plate was used for the cytotoxicity assay. After cell counting, 10,000 cells/well were seeded. After 24 h of incubation, SH-SY5Y cells were treated with 1,25(OH)_2_D_3_ (VEM, Turkey) at doses of 0, 5, 10, 25, 50, 50, 100, 250, 500, and 1,000 nM and incubated with these doses for 24 h. The optical densities were recorded at a wavelength of 570 nm using an automated multi-well plate reader (Synergy HTX, BioTek, USA).

### Wound healing assay

In order to evaluate the impact of 1,25(OH)_2_D_3_ on cellular migration, a wound healing assay was conducted on SH-SY5Y cells in accordance with the following procedure: 3×10^4^ cells/well were seeded on a 6-well plate. To allow the cells to adhere to the surface and form confluent monolayers, they were incubated at 37°C in 5% CO_2_ for 24 h. The cells were scraped vertically with a 200 μL sterile pipette tip. Subsequently, the cells were washed with phosphate-buffered saline in order to remove any residual cell debris. The dose groups were administered doses of 100 and 265.6 nM, while the control group was treated with the culture medium. The wound size was visualised using an inverted microscope, and the wound closure rate was calculated using the ImageJ software, which is a digital image processing and analysis software^7^.

### Real time polymerase chain reaction analysis

Effects of 1,25(OH)_2_D_3_ on mRNA expression analysis in SH-SY5Y cells were performed by the mRNA expression analysis method described previously^8^.

Briefly, 3×10^4^ cells/well were seeded on a 6-well plate. To allow the cells to adhere to the surface and form confluent monolayers, they were incubated at 37°C in 5% CO_2_ for 24 h.

The dose groups were treated with doses of 100 and 265.6 nM for 24 h, while the control group was treated with culture medium. The cycle threshold values of the target genes were determined, and the relative expression levels were calculated using the Livak method, which employs formula 2^-^ΔΔ^Ct9^. The examination of each sample was conducted in triplicate. In Table 1, the primer sequences used for the genes are given.

**Table 1:** Primary sequences list.

Genes	Primary sequence
ACTB	F: CATGTACGTTGCTATCCAGGC
	R: CTCCTTAATGTCACGCACGAT
DNMT3A	F:TATTGATGAGCGCACAAGAGAGC
	R: GGGTGTTCCAGGGTAACATTGAG
DNMT3B	F: GGCAAGTTCTCCGAGGTCTCTG
	R: TGGTACATGGCTTTTCGATAGGA
PTEN	F: CGACGGGAAGACAAGTTCAT
	R: AGGTTTCCTCTGGTCCTGGT

F: forward; R: reverse; ACTB: actin beta; DNMT3A: DNA methyltransferase 3 alpha; DNMT3B: DNA methyltransferase 3 beta; PTEN: phosphatase and tensin homolog.

### Statistical analysis

The statistical analysis was conducted using GraphPad Prism v.9 (San Diego, CA, USA). The differences between the two groups were evaluated using the Student's t-test and one-way analysis of variance (Tukey). A p-value of less than 0.05 was considered statistically significant.

## RESULTS

### MTT assay

A series of 1,25(OH)_2_D_3_ concentrations (0, 5, 10, 25, 50, 100, 250, 500, and 1,000 nM) were subjected to examination. The viability of SH-SY5Y cells decreased in a concentration-dependent manner and was determined as IC_50_:265.6 nM (R^2^: 0.695).

### Wound healing assay

**Figure 1:**
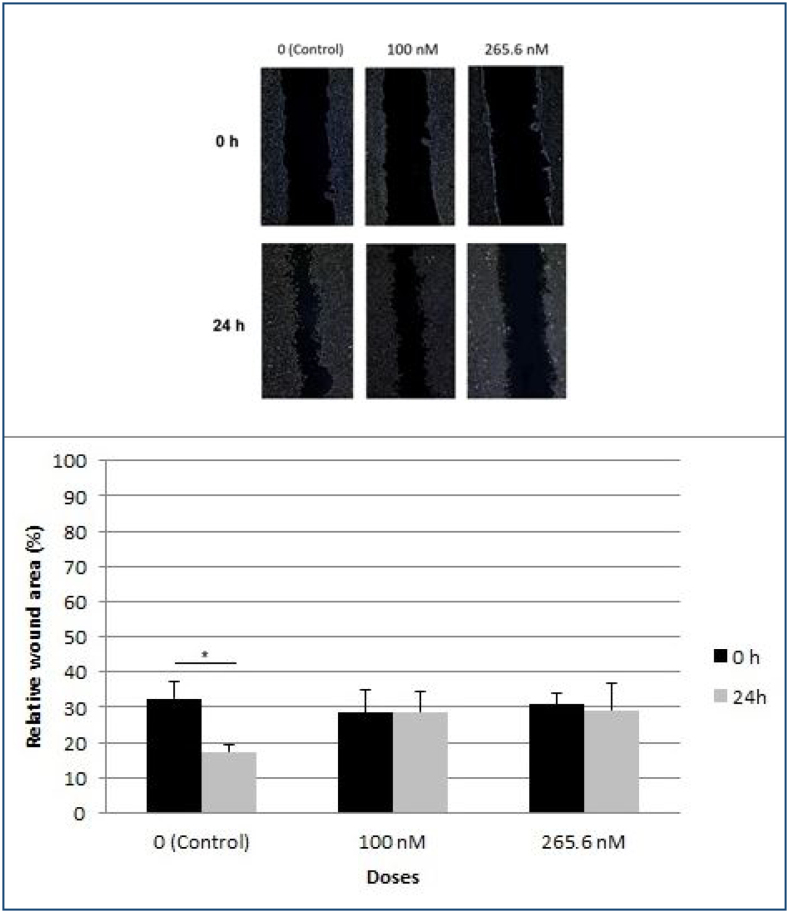
Relative wound area.

The wound-healing assay employed SH-SY5Y cells. Figure 1 illustrates the relative wound area in distinct groups at 0 and 24 h. The 1,25(OH)_2_D_3_ treatment demonstrated a dose-dependent inhibition of the closure of the scratch area. These findings indicate that 1,25(OH)_2_D_3_ impedes cell migration. 100 and 265.6 nM doses showed an antiproliferative effect compared to the control group (p<0.05).

### The results of the mRNA expression analysis

**Figure 2:**
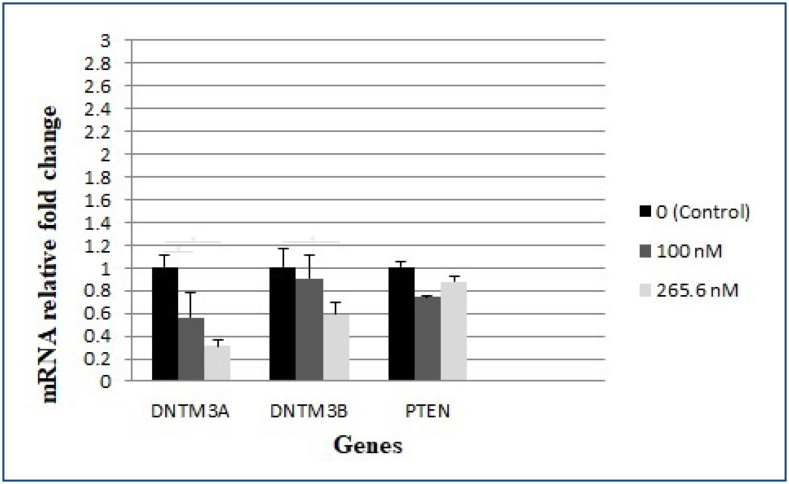
DNMT3A, DNMT3B and PTEN expression fold change.

The expression levels of genes associated with DNA methylation and TSGs were evaluated and compared between groups to gain insight into the underlying mechanisms of tumour formation. The decrease in DNA methyltransferase 3 alpha (DNMT3A) expression level was statistically significant at 100 and 256.6 nM concentration levels compared to the control group (p<0.05). In the DNA methyltransferase 3 beta (DNMT3B) expression level, it was observed that the concentration level of 256.6 nM expressed a statistical significance compared to the control group (p<0.05). In phosphatase and tensin homolog (PTEN) expression levels, there was no statistical significance between any groups (p>0.05) (Figure 2).

## DISCUSSION

Neuroblastoma is a common tumour in the paediatric age group. It is characterised by a complex management regimen and a higher relapse rate in the post-consolidation phase. The presence of vitamin D receptor (VDR) in neuroblastoma cell lines, along with its quantities and the impact of the active vitamin D3 metabolite on VDR signalling, have all been linked to the mechanism of action of vitamin D3 in neuroblastoma cell lines. Vitamin D3 has been demonstrated to exert an antiproliferative effect on a number of different types of cancerous cells, with this effect being achieved through the process of apoptosis, or programmed cell death^10^.

The 3-(4,5-Dimethylthiazol-2-yl)-2,5-Diphenyltetrazolium Bromide (MTT) tetrazolium reduction assay is the inaugural homogeneous cell viability assay that has been developed to be compatible with a 96-well format, thus rendering it suitable for utilisation in high-throughput screening^11^. The antitumour effects of vitamin D3 have been investigated in a range of tumour types using the MTT assay^12^. In an MTT assay of a study in ECV-304 (human urinary bladder carcinoma) and T24 (human bladder carcinoma) cells, a combination treatment of 500 nM vitamin D+16.6 μM cisplatin was compared with cisplatin alone. The results of this experiment showed that only in the T24 cell line did the combination of vitamin D+cisplatin increase antiproliferative activity compared to cisplatin^13^.

Vitamin D3 is believed to exert its protective effects by promoting apoptosis, inhibiting tumour angiogenesis, and regulating antiproliferative, antimetastatic, and prodifferentiation mechanisms in colorectal cancer^14^, breast cancer^15^, prostate cancer^16^, lung cancer^17^, and neuroblastoma cancer cells^10^.

In this study, it was observed that 1,25(OH)_2_D_3_ was effective on SY-SH5Y cell viability and antiproliferative mechanisms with increasing doses, and 100 and 265.6 nM doses were effective on cell migration compared to the control group.

The process of DNA methylation represents an epigenetic mechanism that regulates gene expression. There is mounting evidence that vitamin D3 may play an important role in this regulatory process^18^. Among the epigenetic mechanisms that affect the activity and functionality of specific DNA regions, DNA methylation is provided by DNMTs to direct the transcriptional activity of genes without changing the DNA sequence. DNMTs that affect genomic methylation patterns are DNMT1, DNMT3, DNMT3a, and DNMT3b^19^.

The inactivation of TSGs represents a pivotal mechanism underlying the pathogenesis of all common forms of human cancer^20^. A study has demonstrated that Schisandrin B, an active substance, has been demonstrated to inhibit Aβ1-42-mediated damage in the SH-SY5Y neuronal cell line. Furthermore, this inhibition has been found to significantly suppress the induced alterations in mRNA and protein expression of DNMT3A and DNMT3B, with the concentration of the active substance exerting a significant impact on this process^21^. The depletion of DNMT3A was observed to result in demethylation of the PTEN promoter in hepatocellular carcinoma (HCC) cells, thereby indicating that DNMT3A silences PTEN through DNA methylation. This result provides insight into the mechanisms by which DNMT3A regulates TSGs in HCC through an epigenetic approach^22^. Imatinib has been demonstrated to induce epigenetic alterations of the PTEN gene in leukaemia cells, a process that is mediated by the upregulation of DNMT3A^23^. In another study, methyltransferase DNMT3B was identified as a factor that stimulates the proliferation of colorectal cancer cells^24^. A study has demonstrated that DNMT3A interacts directly with p53, which results in the suppression of p53-mediated transactivation of the p21 gene^25^.

In this study, it was observed that 1,25(OH)_2_D_3_ altered the expression levels of DNMT3A and DNMT3B, but did not cause any change in PTEN expression level in SH-SY5Y cells. These results suggest that the methyltransferase effect of DNMT3A and DNMT3B may be effective on other TSGs and different signalling pathways associated with these genes, but not on PTEN.

## CONCLUSION

Within the limits of this study, the fact that 1,25(OH)_2_D_3_ showed antiproliferative, antimigratory activity on neuroblastoma SH-SY5Y cells and decreased the expression levels of genes involved in methylation may be evidence of its anticancer effect. In this respect, further and more detailed investigations, both in vitro and in vivo, should be performed to strengthen the evidence of the anticancer effect of 1,25(OH)_2_D_3_ in human neuroblastoma.
